# Gut microbiota in a mouse model of obesity and peripheral neuropathy associated with plasma and nerve lipidomics and nerve transcriptomics

**DOI:** 10.1186/s40168-022-01436-3

**Published:** 2023-03-15

**Authors:** Kai Guo, Claudia Figueroa-Romero, Mohamed Noureldein, Lucy M. Hinder, Stacey A. Sakowski, Amy E. Rumora, Hayley Petit, Masha G. Savelieff, Junguk Hur, Eva L. Feldman

**Affiliations:** 1 Department of Neurology, University of Michigan, Ann Arbor, MI 48109 USA; 2 Reata Pharmaceuticals, Irving, TX 75063 USA; 3 Department of Neurology, Columbia University, New York, NY 10032 USA; 4 Department of Biomedical Sciences, School of Medicine and Health Sciences, University of North Dakota, Grand Forks, ND 58202 USA

**Keywords:** Peripheral neuropathy, Dysbiosis, Lipids, Microbiome, Obesity, Omics, Prediabetes

## Abstract

**Background:**

Peripheral neuropathy (PN) is a common complication in obesity, prediabetes, and type 2 diabetes, though its pathogenesis remains incompletely understood. In a murine high-fat diet (HFD) obesity model of PN, dietary reversal (HFD-R) to a low-fat standard diet (SD) restores nerve function and the nerve lipidome to normal. As the gut microbiome represents a potential link between dietary fat intake and nerve health, the current study assessed shifts in microbiome community structure by 16S rRNA profiling during the paradigm of dietary reversal (HFD-R) in various gut niches. Dietary fat content (HFD versus SD) was also correlated to gut flora and metabolic and PN phenotypes. Finally, PN-associated microbial taxa that correlated with the plasma and sciatic nerve lipidome and nerve transcriptome were used to identify lipid species and genes intimately related to PN phenotypes.

**Results:**

Microbiome structure was altered in HFD relative to SD but rapidly reversed with HFD-R. Specific taxa variants correlating positively with metabolic health associated inversely with PN, while specific taxa negatively linked to metabolic health positively associated with PN. In HFD, PN-associated taxa variants, including *Lactobacillus*, *Lachnoclostridium*, and *Anaerotruncus,* also positively correlated with several lipid species, especially elevated plasma sphingomyelins and sciatic nerve triglycerides. Negative correlations were additionally present with other taxa variants. Moreover, relationships that emerged between specific PN-associated taxa variants and the sciatic nerve transcriptome were related to inflammation, lipid metabolism, and antioxidant defense pathways, which are all established in PN pathogenesis.

**Conclusions:**

The current results indicate that microbiome structure is altered with HFD, and that certain taxa variants correlate with metabolic health and PN. Apparent links between PN-associated taxa and certain lipid species and nerve transcriptome-related pathways additionally provide insight into new targets for microbiota and the associated underlying mechanisms of action in PN. Thus, these findings strengthen the possibility of a gut-microbiome-peripheral nervous system signature in PN and support continuing studies focused on defining the connection between the gut microbiome and nerve health to inform mechanistic insight and therapeutic opportunities.

Video Abstract

**Supplementary Information:**

The online version contains supplementary material available at 10.1186/s40168-022-01436-3.

## Background

Obesity, prediabetes, and type 2 diabetes are global epidemics affecting hundreds of millions of people worldwide [1, 2]. Driven by overconsumption of a “Western diet” rich in saturated fats that promote metabolic dysfunction (i.e., glucose intolerance and dyslipidemia), these metabolic conditions are associated with several health complications, including peripheral neuropathy (PN) [3, 4]. PN is defined as distal-to-proximal peripheral nerve damage that results in poor gait, increased risk of foot ulceration and amputation, and lower quality of life [5]. Despite intense research, PN pathogenesis remains incompletely understood, and management remains suboptimal.

The gut microbiome has emerged as a plausible link between dietary intake and metabolic and nerve health. Indeed, a Western diet and high-fat diet (HFD) influence gut microbiota and induce dysbiosis [6–8]. Additionally, obesity and T2D are associated with a perturbed microbial profile [9–11]. In turn, the gut microbiome influences host metabolism by impacting energy utilization, intestinal absorption of macronutrients including lipids, and promoting insulin resistance, hyperglycemia, and dyslipidemia [12–19]. Thus, metabolic symbiosis occurs between the microbiome and host, a relationship modulated by dietary intake.

The microbiome likewise influences nerve health through a microbiome-gut-nervous system axis involving metabolite signaling and the immune system in the context of metabolic dysfunction [20, 21]. Fecal transplant from lean donor mice to recipient animals with HFD-induced obesity reverses small fiber PN and hypersensitivity, accompanied by an improved immune cell profile and an increase in circulating short-chain fatty acids, mainly butyrate [22]. HFD also induces enteric neuropathy by decreasing the density of nitrergic myenteric neurons, changes associated with gut flora restructuring [23]. In humans, T2D patients exhibit a distinct microbiota signature linked to PN status and metabolic status (insulin resistance) [24]. The microbiome may also impact pain in PN through various communication pathways, e.g., immune cells and short-chain fatty acids [22, 25, 26].

Despite several known involved pathways, the precise molecular steps precipitating PN remain elusive. However, the studies indicating potential connections between dietary intake and microbiome structure versus host metabolism and nerve health unlock interesting research avenues. We hypothesize that a microbiome-gut-peripheral nerve axis exists, whereby HFD restructures the gut microbiome which triggers systemic and local metabolic changes that negatively impact peripheral nerve function. This HFD-induced microbiome reorganization and PN relationship suggests that these effects could be reversed through dietary changes. Indeed, we previously demonstrated that dietary reversal (HFD-R) from HFD to a low-fat standard diet (SD) in a HFD obesity mouse model rescues PN phenotypes [27, 28]; however, the impact on the microbiome has not been investigated. Herein, our objective was to leverage SD, HFD, and HFD-R mice to closely examine the microbiome and test the correlations between dietary fat content, gut flora community structure, the plasma and sciatic nerve lipidome, the nerve transcriptome, and PN phenotype in order to gain initial insight into a possible gut-microbiome-peripheral nervous system signature of PN.

## Methods

### Study design, phenotyping, and biospecimen collection

**Fig. 1:**
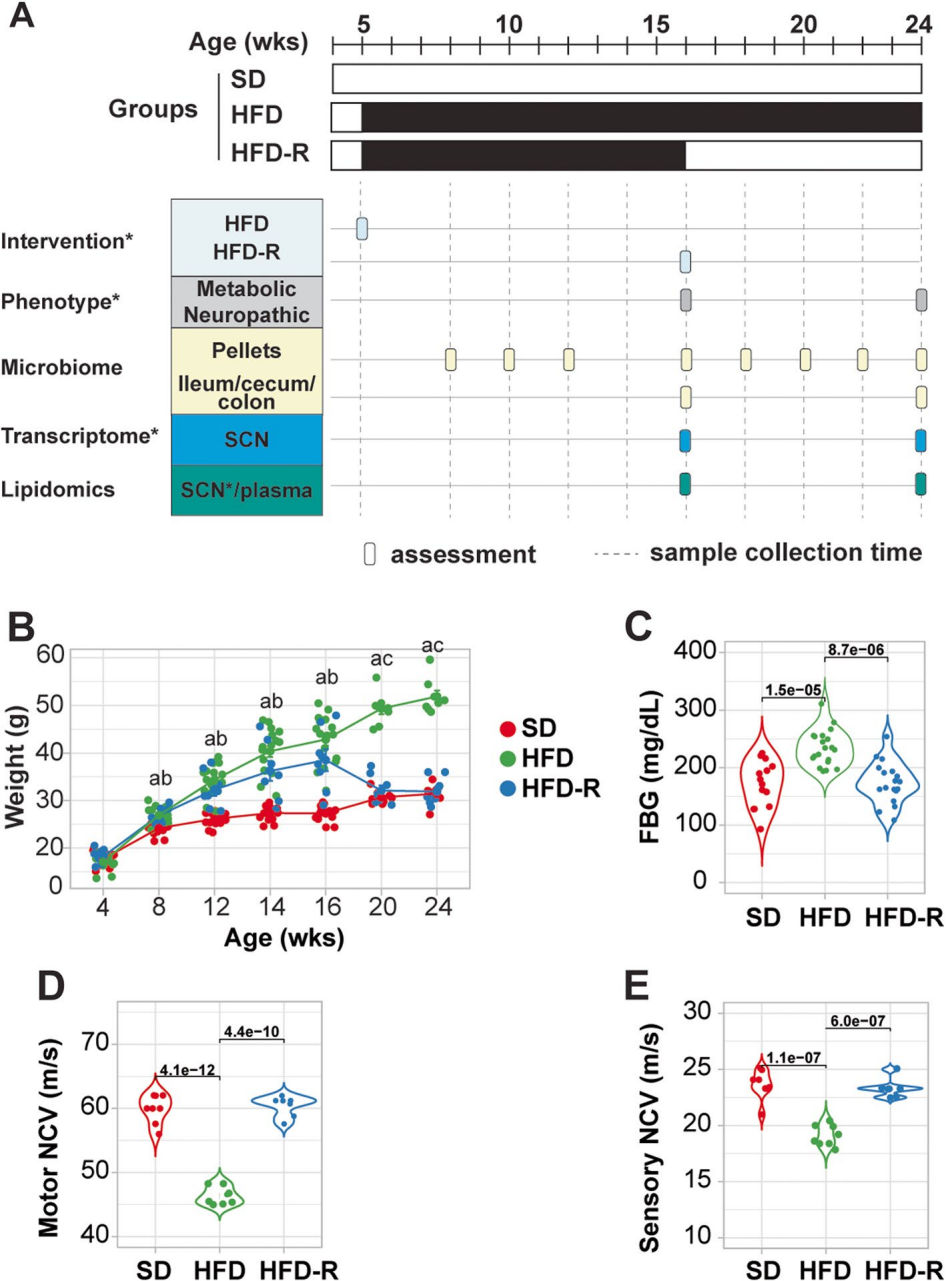
Dietary reversal normalizes metabolic and PN phenotypes in mice. **A** Experimental design depicts standard diet (SD), high-fat diet (HFD), and HFD with dietary reversal (HFD-R) interventions up to 24 weeks (wks) of age in a subset of mice from our larger previously reported study [27]. Asterisks denote datasets from the previous study. **B** and **E** Dietary reversal corrects metabolic and neuropathic phenotypes in HFD mice, including **B** body weight (*n* = 8–16), **C** fasting blood glucose (FBG; *n* = 7–8), **D** motor nerve conduction velocity (NCV; *n* = 7–8), and **E** sensory NCV (*n* = 7–8) at 24 weeks of age. One-way ANOVA followed by Tukey’s post hoc test for multiple group comparisons; a, adjusted *P*-value < 0.05 between HFD versus SD; b, adjusted *p*-value < 0.05 between HFD-R versus SD; c, adjusted *P*-value < 0.05 between HFD-R versus HFD

Mice in the current study (Fig. 1A) represent a subset from a previous larger study [27] that also underwent microbiome assessments and plasma lipidomics. Briefly, a 4-week-old male C57BL/6 J mice (cat. no. 000664, The Jackson Laboratory, Bar Harbor, ME, USA) were split into 3 groups (two groups of *n* = 16/group; one group of *n* = 8/group) and fed SD, deriving 10% kcal from fat (cat. no. D12450B, research diets: 10% kcal fat, 20% Kcal protein, 70% kcal carbohydrate, 3.82% energy density), ad libitum for 1 week to allow habituation. At 5 weeks of age, one group (*n* = 16) was maintained on SD, while the other two groups were switched to HFD, deriving 60% kcal from fat (cat. no. D12492, research diets: 60% kcal fat, 20% kcal protein, 20% kcal carbohydrate, 5.21% energy density). At 16 weeks of age, one HFD group (*n* = 8) underwent dietary reversal (HFD-R) and was switched back to SD for the remainder of the study until 24 weeks of age. These timelines are consistent with established protocols for generation of mouse models with HFD-induced obesity, prediabetes, and PN [29–31]. Mice were maintained and housed at the University of Michigan in a pathogen-free suite following the Committee on Use and Care of Animals guidelines.

All mice underwent metabolic and neuropathy phenotyping at 16 and 24 weeks of age in accordance with guidelines by the Diabetic Complications Consortium (www.diacomp.org), per our standard protocol [27]. Metabolic parameters included body weights (BW) and fasting blood glucose (FBG) levels, while neuropathy phenotyping data consisted of sciatic-tibial motor and sural sensory nerve conduction velocities (NCVs) and analysis of intraepidermal nerve fiber density (IENFD). Additional metabolic and neuropathy phenotype data, including glucose tolerance tests (GTT), oxidized low-density lipoprotein (oxLDL), plasma insulin, cholesterol, and triglyceride lipoprotein profiles, and thermal latency, are previously reported [27]. Fecal pellets were additionally collected directly from animals into sterile Eppendorf tubes at 8, 10, 12, 16, 18, 20, 22, and 24 weeks of age, and data from the 8-, 16-, 18-, and 24-week time points are reported. Mice were sacrificed by lethal pentobarbital (Vortech Pharmaceutical, Dearborn, MI, USA) injection at 16 weeks of age (*n* = 8 SD; *n* = 6 HFD) or 24 weeks of age (*n* = 8 SD, HFD, HFD-R) to collect plasma and content from ~ 3 mm of ileum, cecum, or colon under sterile conditions (Fig. 1A, Table S1).

### Microbiome profiling and analysis

Collected fecal samples and intestinal content were seeded in a PowerMag Glass Bead Plate (MO BIO Laboratories, Carlsbad, CA, USA) to isolate bacterial DNA using a MagAttract PowerMicrobiome DNA/RNA Kit (Qiagen) and epMotion 5075 liquid handling system. Amplification of the V4 region of the bacterial 16S rRNA gene was performed on an Illumina MiSeq at the University of Michigan Microbiome Core, as previously reported [32].

Raw sequencing reads were filtered with the dada2 R package [33] and then de-replicated and de-noised using derepFastq function with default parameters. After building an amplicon sequence variant (ASV) table and removing chimeras, taxonomy was assigned against the SILVA database (v132) [34] natively implemented in dada2. Uncharacterized ASVs that were not assigned to any known species at the phylum level were classified as not assigned (NA). ASVs with NA or appearing in less than three samples were removed using a prevalence threshold < number of samples × 0.05. Alpha diversity within the samples was measured using different metrics implemented in the phyloseq package [35]. Principal coordinate analysis (PCoA) based on Bray–Curtis dissimilarity metrics was performed with the proportional normalized data to reveal differences between various groups or time points. A permutational analysis of variance (PERMANOVA) using the Adonis function as part of the vegan package (https://CRAN.R-project.org/package=vegan) was performed to test the effect of treatment as a continuous variable on group differences.

Differential abundance analysis was performed with the DESeq2 package [36], and significant ASVs were identified with a *p*-value < 0.05. Functional profiling was calculated using the Tax4Fun2 package [37]. Multiple testing of Kyoto Encyclopedia of Genes and Genomes (KEGG) pathway abundance according to sample groups was performed with the most updated KEGG database. Butyrate-producing bacteria were identified by compiling a taxonomy file that contained the major commensal butyrate-producing bacterial species from the literature [38–41]. The discovered ASVs from 16S rRNA sequencing were classified against this curated taxonomy file to identify the butyrate-producing bacteria in our dataset.

### Untargeted and targeted plasma lipidomics

Plasma (*n* = 10/group) collected for this study was analyzed by both untargeted and targeted lipidomics at the University of Michigan Regional Comprehensive Metabolomics Resource Center. Lipids were identified from LC/MS/MS untargeted lipidomics data using the LIPIDBLAST program package (http://fiehnlab.ucdavis.edu/projects/LipidBlast) and quantified using MultiQuant software (AB-SCIEX). Missing values were imputed using a K-nearest neighbor (KNN) algorithm, and data were normalized using internal standards. Lipids were measured in both positive and negative ion modes and then merged by their mean values. Differential lipids were identified by unpaired *t*-test between groups with an adjusted Benjamini–Hochberg *p*-value < 0.05 as the significance cutoff.

Targeted lipidomics was conducted on plasma and sciatic nerve samples collected from the subset of mice (*n* = 10) from a previous larger study [27]. All 10 plasma samples were pooled for a total of 350 µl, and lipids were extracted using organic solvents, as previously reported [42]. Triglycerides were then separated on a thin-layer chromatography plate (Merck, Darmstadt, Germany) using hexane:diethyl ether:acetic acid (80:20:1, v/v), as before [27]. Phospholipids, including sphingomyelin, were separated using a solvent mixture of chloroform:methanol:acetic acid:H_2_O (100:40:12:4, v/v) [43]. Sciatic nerve tissues were pooled, homogenized, and analyzed as described previously [27].

### Previous datasets

Longitudinal metabolic measures, as well as PN phenotyping and sciatic nerve transcriptomic and lipidomic datasets at 16 and 24 weeks of age, were collected from SD, HFD, and HFD-R mice in our previous study [27]. Subsets of these data corresponding to mice also followed for the current study were used for correlation analyses with the newly collected microbiome and plasma lipidomic data.

### Correlation analysis

Spearman’s correlations were calculated between differential abundance of PN-associated ASVs, which overlapped between HFD versus SD and HFD-R versus HFD from the four gut microbiome niches (ileum, cecum, colon, pellets), to metabolic state and PN, sciatic nerve transcriptomics, and plasma and sciatic nerve lipidomics. Pathway analysis using KEGG and Gene Ontology (GO) annotations were performed on correlated genes in the correlation analysis of sciatic nerve transcriptomics to PN-associated ASVs.

### Statistical analysis

All statistical analyses were performed using R software environment (v4.0.1).

## Results

### Dietary reversal normalizes metabolic and PN phenotypes in mice

Here, we employed a HFD mouse model that develops obesity (Fig. 1B; HFD, green) and prediabetes (Fig. 1C; defined by a FBG between 150 and 180 mg/dL) versus SD mice (red). HFD mice also robustly and consistently develop PN, evidenced by slowed motor (Fig. 1D) and sensory (Fig. 1E) NVCs relative to control SD mice. Placing HFD mice at 16 weeks back on SD diet, i.e., HFD-R mice (blue), reverses metabolic and neuropathic deficits. At the 24-week time point, there were no significant differences in FBG (Fig. 1C) or motor and sensory NVCs (Fig. 1 D–E) between HFD-R and SD mice, indicating rescue of metabolic and nerve dysfunction. These data from the current subset of mice parallel those reported for the full larger study cohort [27].

### Dietary reversal shifts microbial structure in obese PN mice

**Fig. 2:**
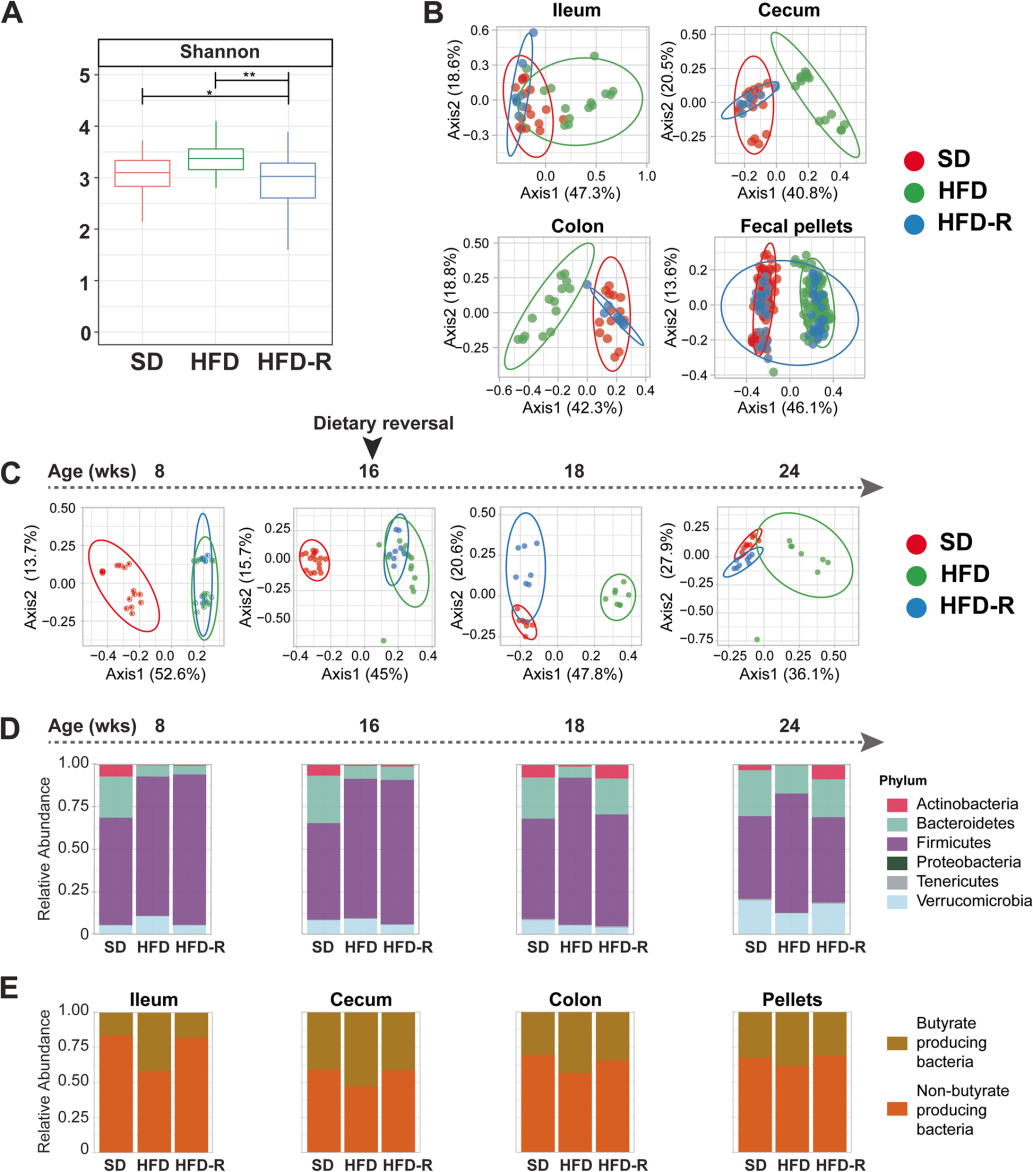
Dietary reversal shifts microbial structure in obese PN mice. **A** Intragroup microbial diversity is significantly higher in HFD (green; *n* = 135) versus SD (red; *n* = 140) and HFD-R groups (blue; *n* = 84) as assessed by alpha diversity using Shannon index. One-way ANOVA; *P < 0.05, **P < 0.01. **B**–**C** Inter-group microbial diversity assessed by beta diversity by ASV clustering by principal coordinate analysis. **B** Differences between gut microbiota community structure in SD (red), HFD (green), and HFD-R (blue) in the ileum, cecum, colon, and fecal pellets. Samples from HFD-R mice prior to dietary reversal from 8 to 16 weeks (wks) of age clustered with HFD samples. All time points are shown (16 and 24 weeks for ileum, cecum, and colon; biweekly samples from 8 to 12 and 16 to 24 weeks for fecal pellets). **C** The microbial community structure in fecal pellets at different time points (8, 16, 18, and 24 weeks of age) rapidly responds to HFD, which is reversed by dietary reversal within a short timeframe (*SD*, *n* = 7–16, red; HFD, *n* = 7–16, green; and HFD-R, *n* = 6–8, blue). **D** Stacked bar plot of the relative abundance of the most abundant taxa at the phylum level in fecal pellets at 8, 16, 18, and 24 weeks of age (SD, *n* = 7–16; HFD, *n* = 7–16; HFD-R, *n* = 6–8). **E** Stacked bar plot of the relative abundance of butyrate-producing bacteria at 24 weeks of age

In our first assessment of microbial community structure, we evaluated intragroup alpha diversity. We observed that alpha diversity was significantly higher in the HFD versus HFD-R and SD versus HFD-R groups, as assessed by the Shannon index in all samples (ileum, cecum, colon, pellets) (Fig. 2A). Examining samples by microbial niche, i.e., ileum, cecum, colon, and pellets, independent of time point, revealed consistent alpha diversity differences, especially marked by the lowest alpha diversity in the small intestine in all diet groups (Figure S1A). When samples were combined by time point and diet, independent of niche, diversity decreased within the SD group at the last time point and within the HDF-R group after dietary reversal at 18 and 24 weeks (Figure S1B); on the other hand, there were no differences in alpha diversity under the HFD intervention.

Next, we examined gut inter-group diversity between samples, assessed by beta diversity of filtered ASVs. Gut microbiome clustered SD from HFD samples, but HFD-R samples following dietary reversal clustered with SD reflecting similar microbial composition (Fig. 2B, Table S2). Longitudinal analysis of fecal pellets indicated microbial communities rapidly adjust to dietary changes (Fig. 2C). Only 3 weeks after mice were initiated on HFD (i.e., 8 weeks of age), there was a distinct shift in microbial community structure versus SD mice (*P* = 0.001). Similarly, dietary reversal for 2 weeks (i.e., 18 weeks of age) already restored altered HFD microbial community structure closer to the SD group (*P* = 0.001). Significant clustering differences between SD and HFD-R versus HFD remained at 24 weeks of age (*P* = 0.001). Notably, although close, SD and HFD-R remained individually clustered at 24 weeks of age. Ileum, cecum, and colon microbiota showed similar shifts in beta diversity between 16 and 24 weeks (Table S2).

Dietary fat content also altered the relative abundance of the most abundant bacterial phyla, which included Actinobacteria, Bacteroidetes, Firmicutes, Proteobacteria, Tenericutes, and Verrucomicrobia, in all gut microbiota niches investigated. Specifically, we observed a decrease in Actinobacteria, Bacteroidetes, and Tenericutes under HFD and a concomitant increase in Firmicutes at all time points, which was reversed by dietary reversal (Fig. 2D). At 24 weeks of age, Proteobacteria was drastically lower in the cecum, colon, and fecal pellets of HFD mice, but was not reversed to SD levels in HFD-R samples. Interestingly, we observed increased butyrate-producing bacteria under HFD compared to SD or HFD-R at 24 weeks of age in the small and large intestines (Fig. 2E). However, analysis of HFD-associated butyrate-producing bacterial composition shows that most are pathogenic species.

### Dietary reversal shifts microbial taxa signature in obese PN mice

**Fig. 3:**
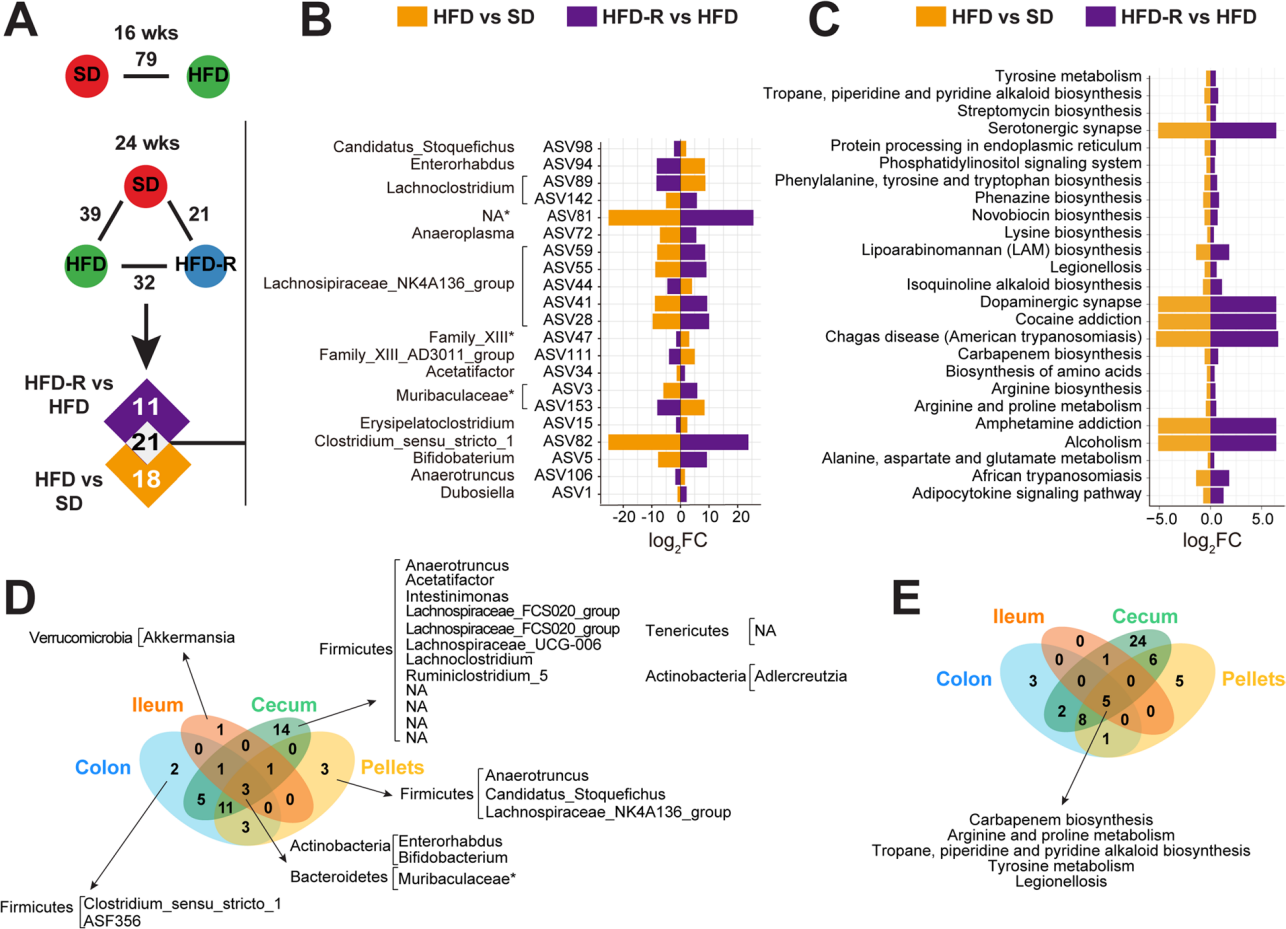
Dietary reversal shifts microbial taxa signature in obese PN mice. **A** Analysis to identify differentially abundant fecal pellet bacteria with DESeq2 between HFD versus SD at 16 weeks of age or between HFD versus SD, HFD-R versus HFD, or HFD-R versus SD at 24 weeks of age (adjusted *p*-value < 0.05). **B** Overlap between gut microbial mediators in fecal pellets driving differences between dietary fat at 24 weeks (HFD versus SD, orange; HFD-R versus HFD, purple) represented as a bar plot of log_2_ fold change (log_2_FC). ASVs are listed with corresponding genus or family (*) level. **C** Overlap between KEGG pathway (level 1) mediators in fecal pellets driving differences between dietary fat at 24 weeks (HFD versus SD, orange; HFD-R versus HFD, purple) represented as a bar plot of log_2_FC. **D**–**E** Venn diagrams of unique and common (**D**) bacterial taxa and **E** KEGG pathways, which change with dietary fat intake in the mouse gut. Multiple *t*-testing was used for pathway comparisons, and significant pathways were identified by FDR-adjusted *p*-value < 0.05. ASV, amplicon sequence variant; wks, weeks

To identify the microbial mediators driving dietary fat-induced changes in gut microbial differences in mice, we identified the most highly differential ASVs in ileum, cecum, colon, and fecal pellets at 16 weeks for HFD versus SD and at 24 weeks for HFD versus SD and HFD-R versus HFD (*P* < 0.05) (Fig. 3A, Figure S2). We then identified taxa signatures that were restored upon changes in dietary fat composition, i.e., dietary reversal, at 24 weeks of age (overlap between HFD versus SD and HFD-R versus HFD). There were 6 ASVs in the ileum, 35 in the cecum, 25 in the colon (Figure S2), and 21 in the fecal pellets (Fig. 3B). These taxa were defined as diet-sensitive ASVs and were used for subsequent analyses in the study. Although each microbiota niche had a unique signature at 24 weeks of age, we identified two ASVs at the genus level, *Enterorhabdus* (ASV94) and *Bifidobacterium* (ASV5) of the phylum Actinobacteria, and one ASV at the family level, Muribaculaceae (ASV3) of the phylum Bacteroidetes, as shared gut bacteria sensitive to dietary fat (Fig. 3D).

Changes in functional gut microbiota composition between dietary interventions in 24-week-old mice were determined by KEGG enrichment analysis. We first identified statistically significant enriched pathways (false discovery rate [FDR] < 0.05) between HFD versus SD and HFD-R versus HFD. We identified 6 overlapping KEGG pathways in the ileum, 46 in the cecum, 19 in the colon (Figure S2), and 25 in the fecal pellets (Fig. 3C). The altered functional categories across microbial niches were mainly related to amino acid biosynthesis and metabolism, insulin resistance/secretion, adipocytokine signaling, bile secretion, and neurotransmitters. The microbiota consistently altered throughout the gut and shared by all niches were the KEGG biological pathways of tyrosine metabolism, arginine and proline metabolism, carbapenem biosynthesis, tropane, piperidine, and pyridine alkaloid biosynthesis, and legionellosis (Fig. 3E).

### Diet-sensitive gut bacteria correlate with metabolic state and PN

**Fig. 4:**
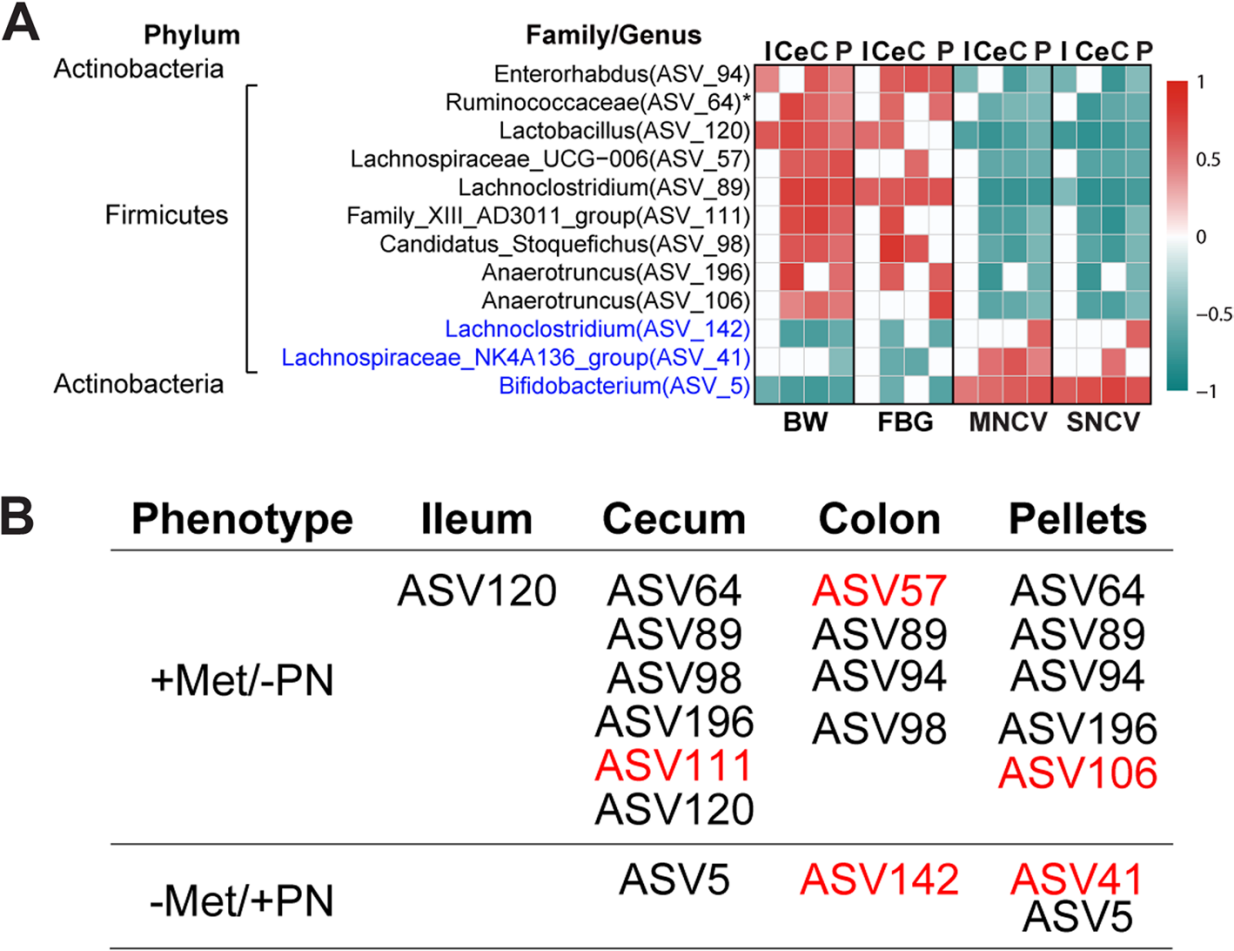
Diet-sensitive gut bacteria correlate with metabolic state and PN at 24 weeks of age. **A** Spearman’s correlation heatmap between gut microbial relative abundance at the genus level with metabolic phenotypes [body weight (BW; *n* = 22–24), fasting blood glucose (FBG; *n* = 14)] and nerve function [motor nerve conduction velocity (MNCV; *n* = 21–23), sensory NVC (SNCV; *n* = 21–23)]. Only ASVs correlating with all parameters in at least one gut niche are shown. ASVs are listed with corresponding genus or family (*) level. Blue ASVs are lower with HFD and correlate negatively with metabolic parameters but positively with PN. Black ASVs are higher with HFD and correlate positively with metabolic phenotypes but negatively with PN. **B** Summary for (**A**); + Met/ − PN, correlates positively with metabolic parameters and negatively with PN parameters; − Met/ + PN, correlates negatively with metabolic parameters and positively with PN parameters. Red ASVs are unique for the specified tissues; black ASVs are shared in different tissues. ASV, amplicon sequence variant; C, colon; Ce, cecum; I, ileum; P, pellet

To correlate the relative abundance of microbiota associated with dietary fat to PN features, we performed correlation analysis (*FDR* < 0.05) of the diet-sensitive ASVs to metabolic (BW, FBG) and PN (motor and sensory NCVs) phenotyping at 24 weeks of age (Fig. 4). Unique and common correlation patterns were observed between ASVs and phenotyping among the ileum, cecum, colon, and fecal pellets. Overall, taxa correlated with metabolic and neuropathic phenotypes in opposite directions, i.e., increased metabolic parameters with decreased nerve function and decreased metabolic parameters with increased nerve function. When screening for ASVs correlating with all parameters in at least one gut niche, we found nine ASVs had a positive correlation to metabolic measurements (BW, FBG) and negative correlation with nerve function (motor and sensory NCVs), and they increased with HFD and decreased with SD (Fig. 4). The ASVs correspond to the genera *Family_XII_AD3011_group* (ASV111), *Lachnospiraceae_UCG-006* (ASV57), *Lachnoclostridium* (ASV89), *Anaerotruncus* (ASV106, ASV196), *Enterorhabdus* (ASV94), *Lactobacillus* (ASV120), and *Candidatus_Stoquefichus* (ASV98) and the family Ruminococcaceae (ASV64). On the other hand, three ASVs, including *Lachnoclostridium* (ASV142), *Lachnospiraceae_NK4A136_group* (ASV41), and *Bifidobacterium* (ASV5), decreased in HFD and increased in SD mice and correlated positively with healthy metabolic and nerve phenotypes.

### Microbial communities associate with plasma and nerve lipidomics and transcriptomics

**Fig. 5:**
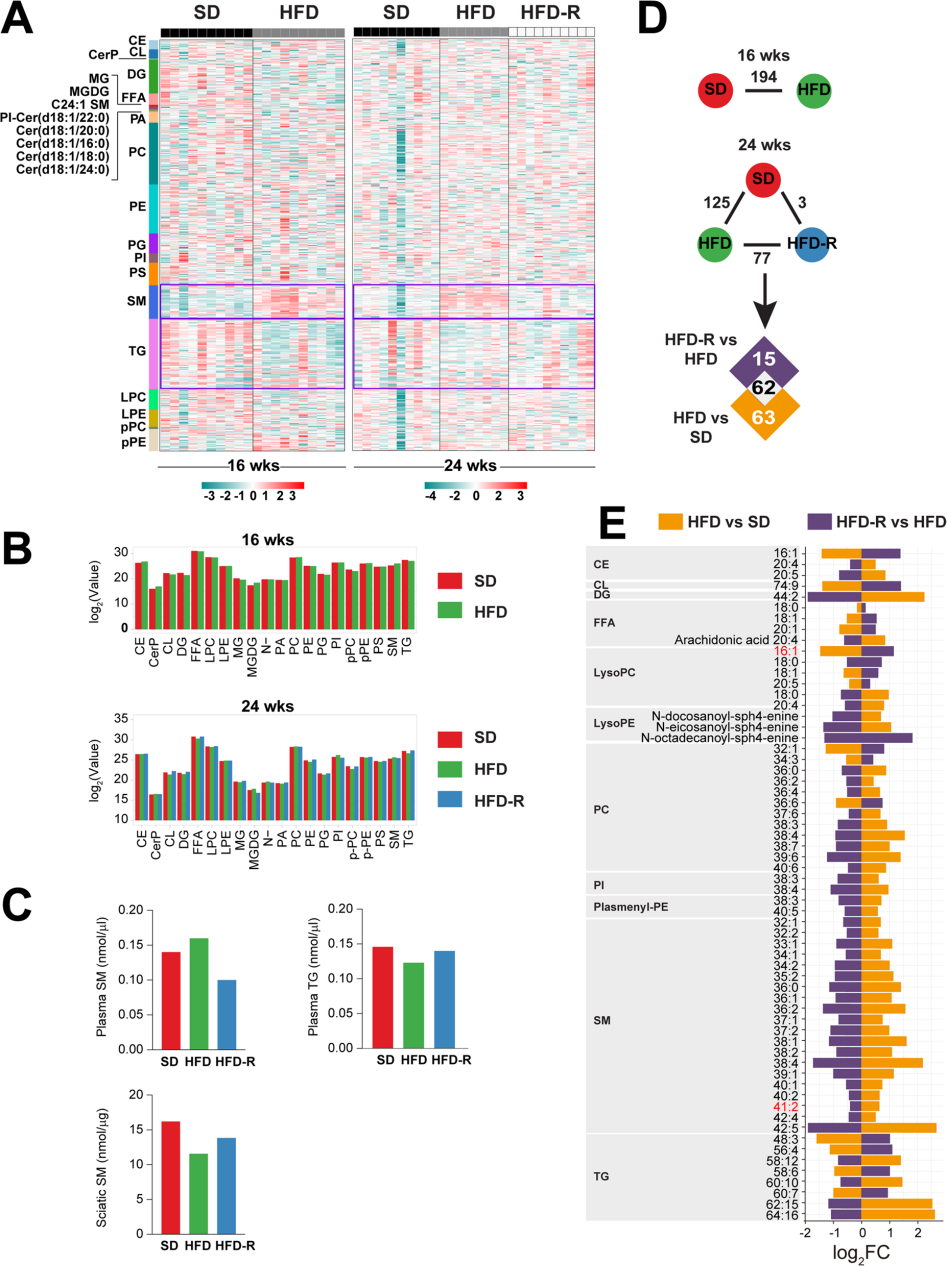
Plasma lipidomics in an obesity PN mouse model and upon dietary reversal. **A** Clustering of lipid classes identified by untargeted lipidomics of plasma from 16- (left, *n* = 20) to 24 (right, *n* = 28)-week-old SD, HFD, or HFD-R mice represented in heatmaps. Purple rectangles outline areas with the most striking differences. **B** Levels of each lipid species from (**A**) were Z-score transformed to generate lipid class aggregates from plasma at 16 (top) and 24 (bottom) weeks of age, represented in bar plots of log_2_(value). **C** The sum of plasma and sciatic nerve (SCN) [27]-targeted lipidomics (TLC-GC) shows higher sphingomyelins and lower triglycerides versus sciatic nerve [27] from HFD compared to SD and HFD-R animals at 24 weeks of age (*n* = 10 plasma samples; *n* = 10 sciatic nerves). **D** Analysis to identify differentially altered lipids (DALs) between HFD versus SD at 16 weeks of age or between HFD versus SD, HFD-R versus HFD, and SD versus HFD-R at 24 weeks of age (adjusted *P* < 0.05). **E** Overlapping DALs from (**D**) show direction of change for HFD versus SD (orange) and HFD-R versus HFD (purple), represented in a bar plot of log_2_ fold change (log_2_FC). Shared lipid species between plasma and sciatic nerve are listed in red text. CE, cholesteryl esters; CL, cardiolipins; CerP, N-hexadecanolsphingosine 1-phosphate; DG, diglycerides; FFA, free fatty acids; MG, 1-acyl-sn-glycerol; MGDG, monogalactosyldiacylglycerol; C24:1 SM, N-15Z-tetracosenoyl-sphing-4-enine; PI-Cer(d18:1/22:0), N-docosanoyl-sphing-4-enine; Cer(d18:1/20:0), N-eicosanoyl-sphing-4-enine; Cer(d18:1/16:0), N-hexadecanoyl-sphing-4-enine; Cer(d18:1/18:0), N-octadecanoyl − sphing-4-enine; Cer(d18:1/24:0), N-tetracosanoyl-sphing-4-enine; PA, phosphatidic acids; PC, phosphatidylcholines; PE, phosphatidylethanolamines; PG, phosphatidylglycerols; PI, phosphatidylinositols; PS, phosphatidylserines; SM, sphingomyelins; TG, triglycerides; LPC, lysophosphatidylcholines; LPE, lysophosphatidylethanolamines; pPC, plasmenyl-phosphatidylcholines; pPE, plasmenyl-phosphatidylethanolamines

Next, we were interested in the correlation between long-term systemic and nerve hyperlipidemia to PN and diet-sensitive microbiota. To address this, we performed untargeted lipidomics on plasma that had been banked from a larger cohort of animals whose sciatic nerves were previously analyzed by lipidomics [27]. In plasma, we identified 578 lipids (25 major lipid class) from 76 samples. Lipid-class-based clustering visualized by heatmaps shows a clear increase in sphingomyelins in HFD versus SD mice in plasma at 16 and 24 weeks of age (Fig. 5A). This pattern was reversed in the HFD-R 24-week-old mice which had undergone dietary reversal. On the contrary, plasma triglycerides decreased in the HFD groups but increased after the mice underwent dietary reversal to SD. These observations were confirmed by lipid class aggregates (Fig. 5B) and targeted lipidomics analysis in a separate cohort of animals under the same dietary paradigm (Fig. 5C). The findings in plasma are opposite to what we previously observed in fat surrounding sciatic nerve; in nerve, triglycerides were elevated in HFD but dropped in response to dietary reversal [27], while sphingomyelins were lower in HFD and increased upon reversal to SD (Fig. 5C).

Individual circulating differentially altered lipids (DALs) were identified by comparing HFD to SD and HFD-R to HFD in 24-week-old mice (Fig. 5 D–E). The levels of 62 DALs changed direction upon dietary reversal; 47 increased and 15 decreased in HFD versus SD, which was reversed in HFD-R versus HFD groups. Lipid species belonging to diglycerides, lysophosphatidylethanolamines, phosphatidylinositols, plasmenyl-phosphatidylethanolamines, and sphingomyelins all increased under HFD and decreased when the animals were switched from HFD to SD.

**Fig. 6:**
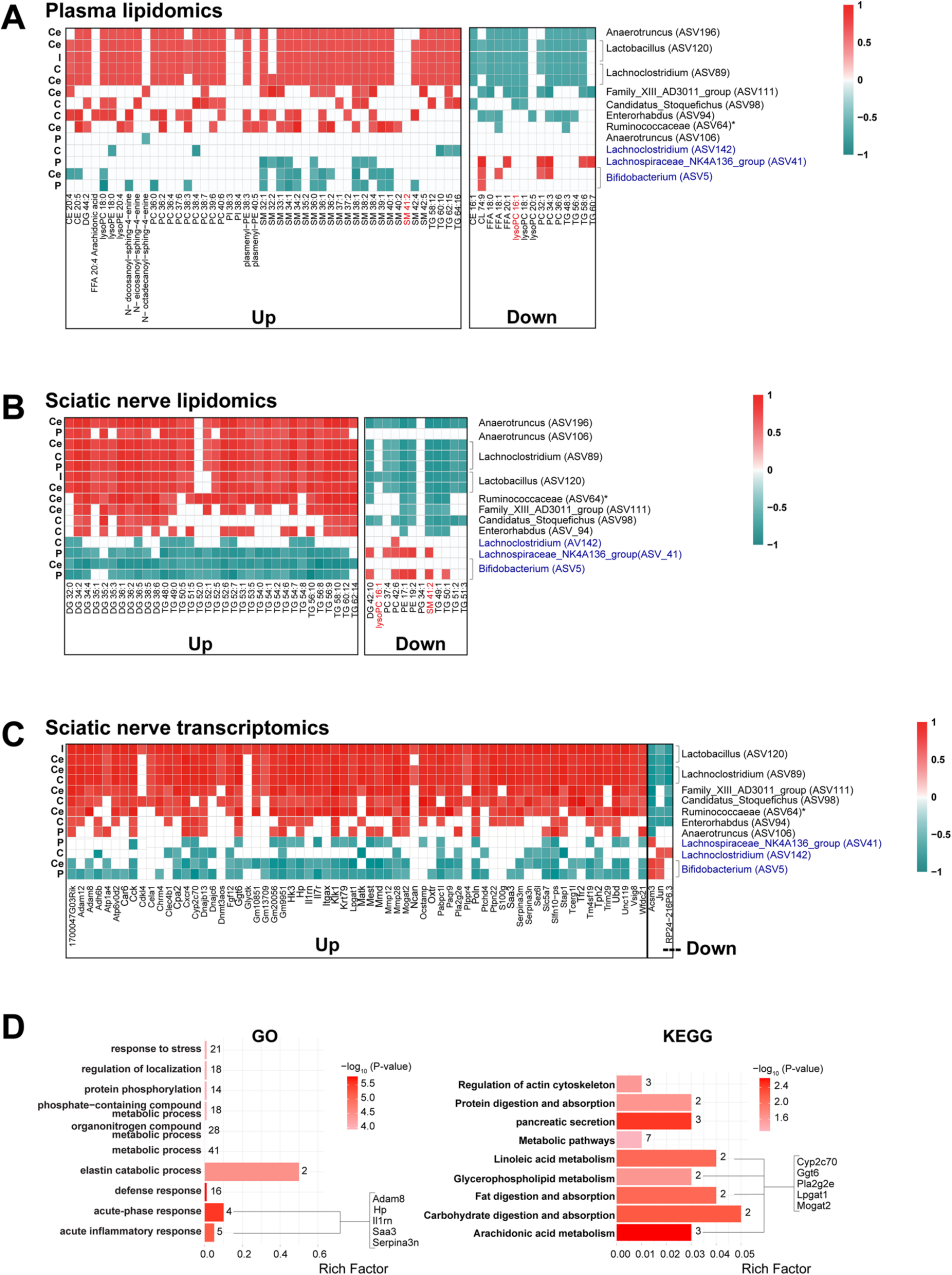
Microbial communities associate with plasma and nerve lipidomics and transcriptomics. **A**–**C** Spearman’s correlation analysis heatmaps (*FDR* < 0.05) of relative abundance of PN-associated gut microbiota sensitive to dietary fat at 24 weeks of age with **A** plasma differentially altered lipids (DALs) (*n* = 11 animals), **B** sciatic nerve DALs [27] (*n* = 11 animals), and **C** sciatic nerve differentially expressed genes (DEGs) [27] (*n* = 11 animals), which are increased (up) or decreased (down) in HFD versus SD. ASVs at the genus or family (*) level that are higher in HFD are listed in black, and ASVs higher in SD are listed in blue. Correlation scale (red, positive; green, negative) is the same for (**A**–**C**). **D** Functional enrichment analysis of 64 increasing and 2 decreasing sciatic nerve DEGs [27] correlating with PN-associated bacteria using gene ontology (GO; left) and Kyoto Encyclopedia of Genes and Genomes (KEGG; right) (*P* < 0.05). The top ten biological pathways are shown. Bar plots indicate the proportion of DEGs assigned to each term (rich factor), with number of genes in each category indicated. CE, cholesteryl esters; CL, cardiolipins; DG, diglycerides; FFA, free fatty acids; lysoPC, lysophosphatidylcholines; lysoPE, lysophosphatidylethanolamines; PI-Cer(d18:1/22:0), N-docosanoyl-sphing-4-enine; Cer(d18:1/20:0), N-eicosanoyl-sphing-4-enine; Cer(d18:1/18:0), N-octadecanoyl-sphing-4-enine; PC, phosphatidylcholines; PE, phosphatidylethanolamines; PI, phosphatidylinositols; pPE, plasmenyl-phosphatidylethanolamines; SM, sphingomyelins; TG, triglycerides. ASV, amplicon sequence variant; FDR, false discovery rate; C, colon; Ce, cecum; I, ileum; P, pellets

Next, to determine whether PN-associated bacteria sensitive to dietary fat correlate with altered host lipid profiles, we performed a series of correlation analyses (*FDR* < 0.05). We first determined the correlation between the relative abundance of ASVs identified in Fig. 4 to plasma lipids that changed upon dietary reversal (Fig. 6A; full correlation Figure S3A). Gut bacteria increasing in HFD, such as *Lactobacillus* (ASV120), *Lachnoclostridium* (ASV89), and *Anaerotruncus* (ASV196), directly correlated with many plasma DALs that increased in HFD, especially sphingomyelins. These ASVs showed inverse correlations with lipids that decreased in response to HFD. A similar pattern was observed with *Enterorhabdus* (ASV94), Ruminococcaceae (ASV64), *Family_XIII_AD3011_group* (ASV111), *Anaerotruncus* (ASV106), and *Candidatus stoquefichus* (ASV98), although correlations were limited to fewer lipid species. On the other hand, ASVs that increased with SD, such as *Bifidobacterium* (ASV5) and *Lachnospiraceae_NK4A136_group* (ASV41), correlated positively with decreasing plasma lipids (triglycerides, free fatty acids, phosphatidylcholines) and negatively with increasing lipids (mostly sphingomyelins) in the cecum, colon, and pellets samples (Fig. 6A). *Lachnoclostridium* (ASV142) also negatively correlated with increased lipids (mostly triglycerides) but had no correlation with decreasing lipids.

To determine whether diet-dependent microbial changes also correlate with lipid species surrounding the sciatic nerve at 24 weeks, we performed correlation analysis between the relative abundance of the PN-associated bacteria and sciatic nerve DALs at 24 weeks of age (Fig. 6B; full correlation Figure S3B) [27]. In contrast to plasma lipidomics, triglycerides and diglycerides around the sciatic nerve were higher in HFD and decreased in response to dietary reversal [27]. We observed positive correlations of bacteria species, such as *Lactobacillus*, *Lachnoclostridium*, and *Anaerotruncus*, to elevated triglycerides and diglycerides in HFD sciatic nerve. Sphingomyelin 41:2 and lysophosphatidylcholine 16:1 were the only lipid species that changed with dietary intervention and overlapped between plasma and sciatic nerve at 24 weeks; however, only lysophosphatidylcholine 16:1 changed in the same direction in both tissues (downregulated in HFD animals and upregulated in SD animals) and negatively correlated with *Candidatus stoquefichus* (ASV98), *Lactobacillus* (ASV120), and *Anaerotruncus* (ASV196) in both plasma and sciatic nerve.

Finally, we performed correlation analysis between the relative abundance of PN-associated bacteria to sciatic nerve transcriptomics profile at 24 weeks of age (Fig. 6C; full correlation Figure S4) [27]. *Lactobacillus* (ASV120), *Lachnoclostridium* (ASV89), *Family_XIII_AD3011_group* (ASV111), *Candidatus stoquefichus* (ASV98), Ruminococcaceae (ASV64), and *Enterorhabdus* (ASV94) correlated positively with upregulated genes and negatively with downregulated genes in HFD sciatic nerve. *Bifidobacterium* (ASV5), *Lachnospiraceae_NK4A136_group* (ASV41)*,* and *Lachnoclostridium* (ASV142) correlated negatively with upregulated genes and positively with downregulated genes in HFD sciatic nerve.

GO and KEGG pathway analysis of DEGs that correlated with PN-associated microbiota was enriched in inflammatory response driven by disintegrin and metalloproteinase domain-containing protein 8 (*Adam8*), haptoglobin (*Hp*), interleukin-1 receptor antagonist (*Il1rn*), serum amyloid A 3 (*Saa3*), and serpin family A member 3 (*Serpina3n*) (Fig. 6D). Also, lipid and bile metabolism and antioxidant defense pathways were represented by cytochrome P450, family 2, subfamily c, polypeptide 70 (*Cyp2c70*), gamma-glutamyl transferase 6 (*Ggt6*), phospholipase A2 group IIE (*Pla2g2e*), lysophosphatidylglycerol acyltransferase 1 (*Lpgat1*), and monoacylglycerol O-acyltransferase 2 (*Mogat2*). These genes mostly correlated positively with ASVs upregulated in HFD, indicating inflammatory, lipid and bile metabolism, and antioxidant defense pathways are linked with PN.

## Discussion

In the current study, we leveraged our HFD obesity PN model with a paradigm of dietary reversal to investigate a putative association between the microbiome-gut-peripheral nerve axis and dietary fat intake. We report that even a short duration of high-fat feeding altered microbiome structure in mice, which rapidly reversed when animals were placed back on SD. These changes occurred through microbial ASVs linked to various metabolic and biosynthetic pathways in all four gut niches, i.e., ileum, cecum, colon, and fecal pellets. Correlation analysis further identified specific microbiome signatures linked with metabolic health and nerve function. Correlations between PN-associated ASVs from HFD animals, lipidomics, and transcriptomics data revealed that *Lactobacillus*, *Lachnoclostridium*, and *Anaerotruncus* taxa variants positively correlated with several lipid species, particularly elevated plasma sphingomyelins and sciatic nerve triglycerides. Relationships were also identified between specific PN-associated taxa variants to expression of genes in neuropathic nerves related to pathways involved in PN pathogenesis, including inflammation, lipid metabolism, and antioxidant defense pathways. These data link HFD-mediated PN to the gut microbiome.

Using our HFD mouse model of obesity, prediabetes, and PN and established time points for dietary alteration and phenotyping [29–31], we observed a dynamic gut microbial community structure throughout the intestine that was rapidly reshaped within weeks by dietary fat content. Similar findings are reported in humans and rodents within the same timeframe [44, 45], including in response to a 60% kcal HFD [46]. As previously published [47], alpha diversity was lowest in the ileum across gut niches for all diets, i.e., SD, HFD, and HFD-R, compared to large intestine. Across diets, we found alpha diversity was highest in HFD metabolically unhealthy obese mice versus SD and HFD-R. This likely represents community structure shifts in the HFD microbiome, which increase the diversity of deleterious bacteria [48], as observed in HFD mouse gut or fecal pellets by Shannon index [46, 49]. Indeed, closer examination of the individual bacterial composition of the HFD microbiota revealed increased abundance of bacteria belonging to Lachnospiraceae, Oscillospiraceae, and Clostridiaceae, families which contain pathogenic bacteria [50–54].

When we assessed beta diversity, SD samples clustered separately from HFD samples, indicating distinct microbiome structure. HFD-R samples clustered closely with SD samples, but did not ever fully reverse, in line with previously observed reports [7, 23, 46]. Our results examining phyla abundance linked to diet indicate that HFD promotes Firmicutes and reduces Bacteroidetes, i.e., high Firmicutes/Bacteroidetes ratio. Similarly, HFD enhances the proportion of butyrate-producing bacteria. While elevated Firmicutes/Bacteroidetes ratio and butyrate-producing bacteria are generally linked to a healthful status [11], several studies have noted, like our findings, elevated Firmicutes/Bacteroidetes ratio [7, 23, 46, 55, 56] and butyrate-producing metagenes [49] in HFD. Discrepancies among studies may arise from differences in host genetic background [49, 57] or species of Firmicutes phylum [58]. Additionally, the families of bacteria we observed in our bacterial composition analysis (Lachnospiraceae, Oscillospiraceae, and Clostridiaceae) produce butyrate [59–61] and thus also likely contribute to the observed increase in butyrate-producing bacteria in HFD mice.

We next investigated specific ASVs, which were identified down to the family or genus level. Several ASVs across the four gut niches were sensitive to diet, i.e., differentially abundant in HFD versus SD and HFD-R versus HFD. *Enterorhabdus* and *Bifidobacterium*, of the phylum Actinobacteria, and Muribaculaceae, of the phylum Bacteroidetes, were shared by all gut niches, whereas *Clostridium_sensu_stricto_1* was prominent in colon and fecal pellets. In the literature, *Bifidobacterium* [7, 55, 62] and *Clostridium_sensu_stricto* genera [55] are lower and *Enterorhabdus* [62] higher in mouse HFD gut, although, conversely, various *Clostridium* species, e.g., *Clostridium CAG:58* and *Clostridium orbiscindens*, instead correlate positively with obesity (BMI, visceral fat) [63] and animal-based diet [44] in human microbiome. Thus, species-level differences in diet-induced gut microbial restructuring may be occurring.

We further considered the functional implications of these differential ASV abundances by performing KEGG pathway analysis comparing HFD to SD. Recurrent among the gut niches were tyrosine metabolism, arginine and proline metabolism, carbapenem biosynthesis, tropane, piperidine and pyridine alkaloid biosynthesis, and legionellosis. In the large intestine, the most significant pathway was insulin resistance, whereas in fecal pellets, neurotransmitter pathways and serotonergic and dopaminergic synapse featured prominently. Several studies have shown correlation of insulin resistance with distinct gut microbial communities [15, 16, 56, 63]. Related to insulin resistance, and also represented in the KEGG analyses on cecum, colon, and fecal pellets, were adipokine signaling (same fold-change direction) and insulin secretion (opposite direction). The literature also underscores the relevance of gut microbiome metabolism related to neurotransmitter biosynthesis, e.g., tyrosine, tryptophan, phenylalanine, and glutamate metabolism and serotonin and dopamine signaling, which are central to nervous system health [20].

Although the impact of HFD on gut microbiome structure is well established [7, 23, 44, 46, 49, 55–58, 63], the effect on peripheral nerve health is less investigated. Thus, after analyzing dietary fat content-induced gut flora changes, we assessed the correlation of microbial ASVs with metabolic parameters and PN phenotypes. Generally, HFD increases BW and FBG and decreases motor and sensory NCVs [27, 28], leading to an inverse relationship in these metabolic parameters to PN phenotype. In the current study, nine ASVs correlated positively with metabolic measurements (BW, FBG) and negatively with nerve function and were elevated in HFD and reduced in SD, while three ASVs that associated negatively with metabolic parameters and positively with nerve function were decreased in HFD and increased in SD. Thus, nerve health correlates with distinct microbiome signatures. Other studies have also noted a microbiome signature of PN in humans [24] and enteric neuropathy in mice [23, 55]. Although ASVs were not provided for direct comparison, similar genera to those identified herein, such as *Lactobacillus*, *Bifidobacterium*, and *Lachnoclostridium*, were among those that differentiated enteric neuropathy [23, 55] and PN [24], among others.

To establish a potential link between diet, PN, and microbiome to specific lipid species, we conducted lipidomics analysis of plasma from 24-week-old SD, HFD, and HFD-R mice, which we combined with our published sciatic nerve lipidomics dataset under the same diet paradigms [27]. Our observations for triglycerides and sphingomyelin showing inverse levels between plasma and sciatic nerve lipids, i.e., in HFD versus SD and HFD-R, agree with reports of elevated plasma/serum sphingomyelins in HFD mice [64, 65] and obese humans [66, 67]. In our correlation analysis of PN-associated ASVs with plasma and sciatic nerve lipids, the most salient associations emerged between elevated circulating sphingomyelins and lower triglycerides in HFD mice, which were negatively linked to certain microbiota species in the cecum, colon, and pellets samples. *Bifidobacterium*, a beneficial bacterial genus used in probiotics supplements, correlates with improved gut microbiome structure [68–70]. Additionally, *Bifidobacterium pseudolongum* supplements decrease plasma triglyceride levels in HFD mice [70], which would improve metabolic profile. We found gut bacteria increasing in HFD, such as *Lactobacillus*, *Lachnoclostridium*, and *Anaerotruncus*, correlated negatively with several free fatty acid and complex lipid species, including LysoPC 16:1, in both plasma and sciatic nerve. In type 2 diabetes patients, *Lachnoclostridium* correlated positively with total cholesterol and low-density lipoprotein cholesterol and a poorer metabolic profile [24].

We finally examined microbial correlations to nerve transcriptome. Most PN-associated gut microbiota positively correlated with upregulated sciatic DEGs, although two downregulated DEGs (*Acsm3* and *Jun*) negatively correlated with PN-associated microbiota. *Acsm3* is an important enzyme in butyrate metabolism, as it activates medium-chain fatty acids towards mitochondrial *β*-oxidation [71]. Downregulated *Acsm3* in HFD sciatic nerve may potentially be a compensatory mechanism to slow butyrate metabolism in attempts to maintain nerve butyrate levels [22]. Similarly, we observed downregulated *Jun*, encoding c-Jun, a protein highly expressed in injured Schwann cells [72] and downregulated during myelination in vivo [73].

All other sciatic nerve DEGs correlated positively with gut microbiota and PN phenotypes, indicating an important link between the microbiota and PN. Pathway analysis of the correlated sciatic DEGs in HFD was related to inflammation (included genes *Adam8*, *Saa3*, *Il1rn*, *Serpina3n*) and lipid, bile, and antioxidant metabolism (*Cyp2c70*, *Ggt6*, *Pla2g2e*, *Lpgat1*, *Mogat2*). Notably, *IL1rn* is involved in granulocyte adhesion and is associated with PN in diabetes *db/db* murine models [74], and Saa3 is an inflammatory marker of Schwann cell injury in peripheral nerves [75]. Both *IL1rn* and *Saa3* are markers of sterile inflammation, are modulated by gut microbiota [76, 77], and are associated with PN in both *ob/ob* and *db/db* mouse models [27, 78–80]. Mice with Serpin3 deficiency exhibit neuropathic pain, which can be reversed by exposure to exogenous Serpin3 [81]. We also identified Adam8, which stimulates axonal extension, as a novel target linking the gut microbiota to sciatic PN [82]. Many of the DEGs related to lipid metabolism (*Pla2g2e*, *Lpgat1*, *Mogat2*) have been previously identified in the sciatic nerve of HFD mice and are involved in linoleic acid, phospholipid, and neutral lipid metabolism [27], suggesting a link between gut microbiota and sciatic lipid metabolism.

This study has limitations. First, it was a correlative study, not a causative one, which would require fecal transplants or antibiotic treatment. However, fecal transplants from lean donor mice to recipient mice with HFD-induced obesity reverse PN phenotypes and immune profiles [22], suggesting possible causality between microbiome and PN. Second, our study only involved male mice, which may not identify important findings due to sex differences in lipids [66, 83, 84] and the microbiome [20, 85]. Finally, our HFD model is based on a homogenous mouse C57BL/6 background in an experimental setting. However, genetics influences microbiome [49, 57] and metabolic phenotype [28]; thus, in the real-world setting, intraindividual variation is likely to moderate the relationships identified in this study.

## Conclusions

Overall, we report the presence of a PN-associated microbiome signature in response to dietary fat. In correlation analyses, *Lactobacillus*, *Lachnoclostridium*, and *Anaerotruncus* ASVs positively correlated with several lipid species, particularly elevated plasma sphingomyelins and sciatic nerve triglycerides in HFD mice with PN. In sciatic nerve transcriptome, PN-associated ASVs were linked to gene expression related to inflammation, lipid metabolism, and antioxidant defense, intimating a potential gut-microbiome-peripheral nerve system. These findings underscore the importance of microbiota in PN pathogenesis. The identified HFD-associated microbial species could potentially serve as biomarkers to predict PN susceptibility in obese, prediabetic, and diabetic individuals, and clinical studies are warranted to test the correlation between these microbial species and human PN. Additionally, our assessment of dietary impact on microbiota composition shows that a HFD is associated with pathogenic microbiota, while a SD is associated with more beneficial microbiota. This offers insight into novel therapeutic strategies for PN that focus on diets with low-fat content and beneficial microbiota, supplied either via probiotics or fecal microbial transplant. Importantly, manipulation of microbiota has been successfully applied in obese, prediabetic, and diabetic individuals to improve their health and quality of life [86–90]. Our data support the contention that shifting away from a Western diet can delay PN and identify the microbiome as a potential target for therapeutic intervention in PN. Continuing studies focused on defining the connection between the gut microbiome and nerve health are thus warranted.

## Supplementary Information

**Additional file 1:** Supplementary information: Supplementary figures:** Figure S1.** Alpha diversity across microbiome samples. **Figure S2.** Dietary reversal shifts microbial taxa signature in obese PN mice. **Figure S3.** Full correlation analysis for microbial communities to plasma and sciatic nerve lipidomics. **Figure S4.** Full correlation analysis for microbial communities to plasma and sciatic nerve transcriptomics. Supplementary tables: **Table S1.** Study microbiome samples. **Table S2.** Beta diversity in microbiome samples.

## Data Availability

The raw murine 16S sequencing dataset generated and analyzed in the current study is deposited in NCBI SRA under the BioProject accession number PRJNA700071. All the scripts are available from the corresponding author upon request. Supplementary information is available in Additional file 1.

## Abbreviations

Adam8: Disintegrin and metalloproteinase domain-containing protein 8
ASV: Amplicon sequence variant
C24:1 SM: N-15Z-tetracosenoyl-sphing-4-enine
CE: Cholesteryl esters
Cer(d18:1/20:0): N-Eicosanoyl-sphing-4-enine
Cer(d18:1/16:0): N-Hexadecanoyl-sphing-4-enine
Cer(d18:1/18:0): N-Octadecanoyl − sphing-4-enine
Cer(d18:1/24:0): N-Tetracosanoyl-sphing-4-enine
CerP: N-Hexadecanolsphingosine 1-phosphate
CL: Cardiolipins
Cyp2c70: Cytochrome P450, family 2, subfamily c, polypeptide 70
D: Diglycerides
DALs: Differentially altered lipid
DEG: Differentially expressed genes
FBG: Fasting blood glucose
FFA: Free fatty acids
Ggt6: Gamma-glutamyltransferase 6
GO: Gene ontology
HFD: High-fat diet
HFD-R: High-fat diet reversal
Hp: Haptoglobin
Il1rn: Interleukin-1 receptor antagonist
KEGG: Kyoto Encyclopedia of Genes and Genomes
LP: Lysophosphatidylethanolamines
LPC: Lysophosphatidylcholines
Lpgat1: Lysophosphatidylglycerol acyltransferase 1
MG: 1-Acyl-sn-glycerol
MGDG: Monogalactosyldiacylglycerol
Mogat2: Monoacylglycerol O-acyltransferase 2
NVC: Nerve conduction velocities
PA: Phosphatidic acids
PC: Phosphatidylcholines
PCoAL: Operational taxonomic units
PE: Phosphatidylethanolamines
PG: Phosphatidylglycerols
PI: Phosphatidylinositols
PI-Cer(d18:1/22:0): N-docosanoyl-sphing-4-enine
Pla2g2e: Phospholipase A2 group IIE
PN: Peripheral neuropathy
pPC: Plasmenyl-phosphatidylcholines
pPE: Plasmenyl-phosphatidylethanolamines
PS: Phosphatidylserines
Saa3: Serum amyloid A 3
SD: Standard diet
Serpina3n: Serpin family A member 3
SM: Sphingomyelins
TG: Triglycerides
