# Supplementary material for: Gut microbiota in a mouse model of obesity and peripheral neuropathy associated with plasma and nerve lipidomics and nerve transcriptomics
Source: Microbiome. 2023 Mar 15;11:52. doi: 10.1186/s40168-022-01436-3 (PMC10015923; doi:10.1186/s40168-022-01436-3)
Supplement: Supplementary file 2 — Additional file 1: Supplementary information: Supplementary figures: Figure S1. Alpha diversity across microbiome samples. Figure S2. Dietary reversal shifts microbial taxa signature in obese PN mice. Figure S3. Full correlation analysis for microbial communities to plasma and sciatic nerve lipidomics. Figure S4. Full correlation analysis for microbial communities to plasma and sciatic nerve transcriptomics. Supplementary tables: Table S1. Study microbiome samples. Table S2. Beta diversity in microbiome samples. [file 40168_2022_1436_MOESM1_ESM.pdf]

SUPPLEMENTARY INFORMATION

Gut microbiota in a mouse model of obesity and peripheral neuropathy associate with plasma and nerve lipidomics and nerve transcriptomics

Mohamed Noureldein[1,§], Kai Guo[1,§], Claudia Figueroa-Romero[1,§], Lucy M. Hinder[1,#], Stacey A. Sakowski[1], Amy E. Rumora[1], Hayley Petit[1], Masha G. Savelieff[1], Junguk Hur[2], Eva L. Feldman[1,*]

[1]Department of Neurology, University of Michigan, Ann Arbor, MI 48109, USA

[2]Department of Biomedical Sciences, School of Medicine and Health Sciences, University of North Dakota, Grand Forks, ND 58202, USA

[Φ]Current: Reata Pharmaceuticals, Plano, Texas 75024, USA.

Present address:

#Reata Pharmaceuticals, Irving, Texas 75063, USA.

¶Department of Neurology, Columbia University, New York, NY, 10032, USA

[§]Authors contributed equally

*Address correspondence to:

Eva L. Feldman, MD, PhD

Department of Neurology, University of Michigan

109 Zina Pitcher Place, 5017 AAT-BSRB, Ann Arbor, MI 48109-2200

Phone: 734-763-7269 / Fax: 734-763-7275 / Email: efeldman@umich.edu

SUPPLEMENTARY FIGURES

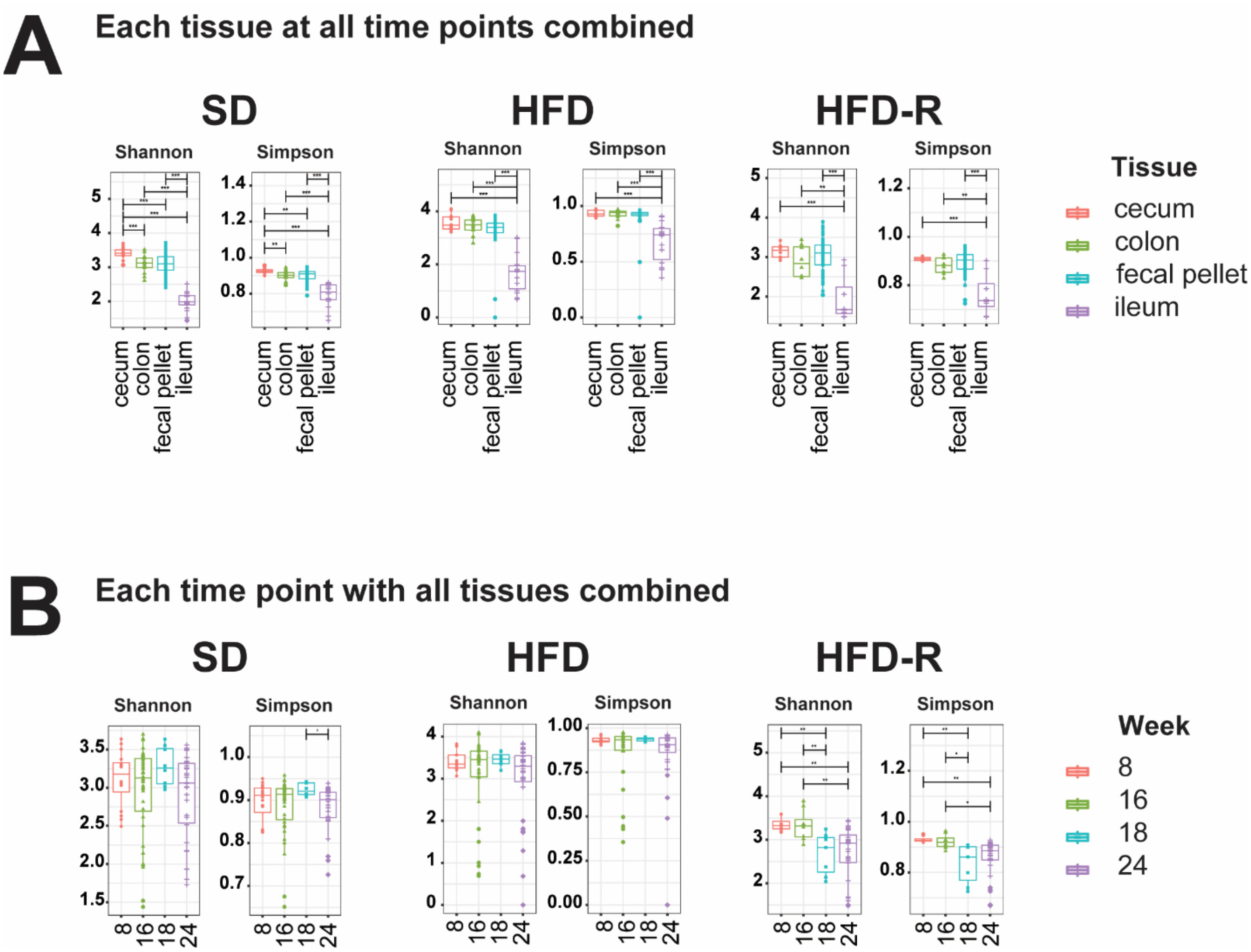

Figure S1. Alpha diversity across microbiome samples. Alpha diversity measured by Shannon and Simpson indexes (**A**) by microbial niche, *i.e.*, ileum, cecum, colon, and fecal pellets, independent of time point, or (**B**) by time point at 8, 16, 18, and 24 weeks of age, independent of microbial niche. One-way ANOVA; *p<0.05, **p<0.01, ***p<0.001.

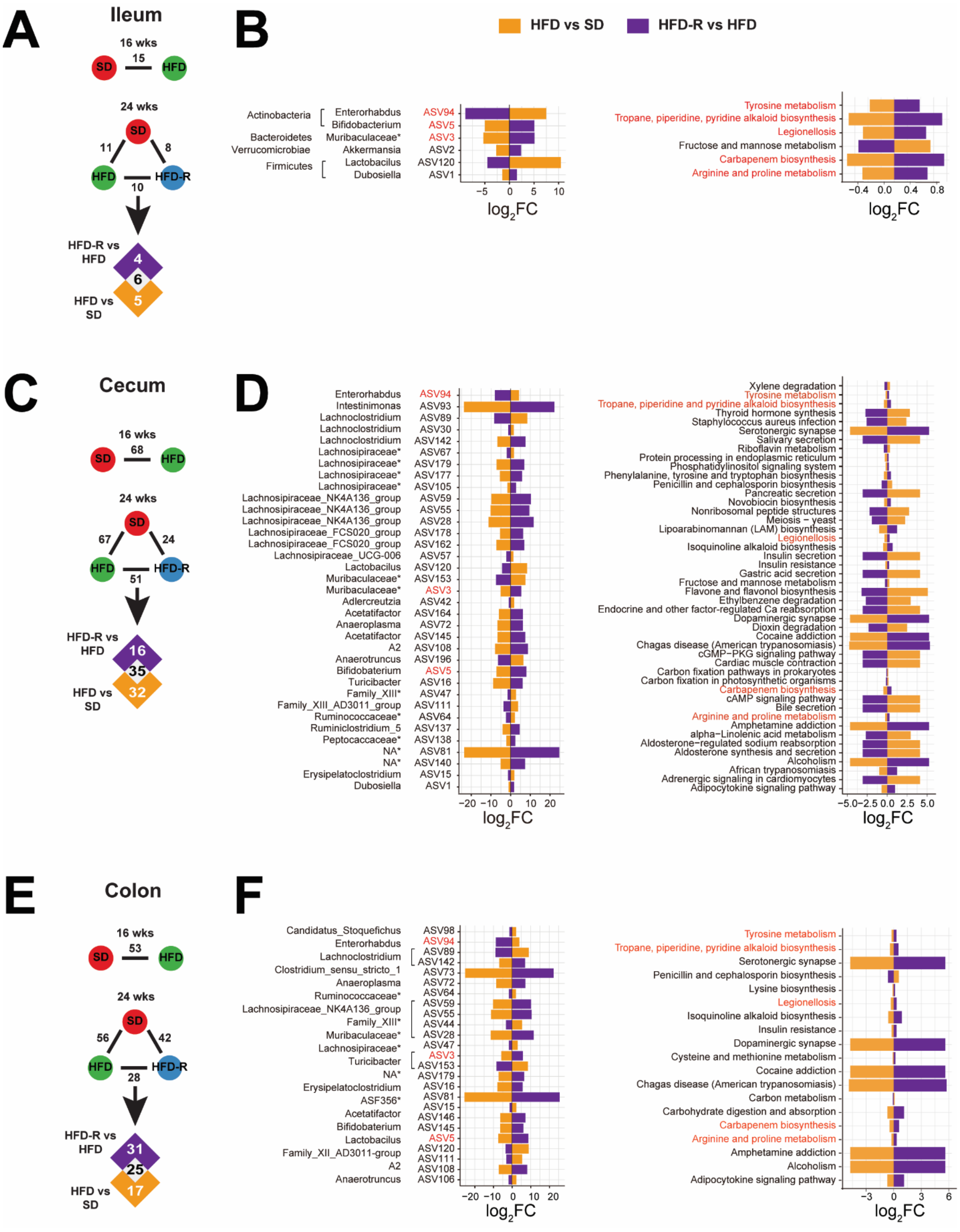

A
Ileum
16 wks
15
SD
HFD
24 wks
11
8
10
HFD-R
HFD-R vs HFD
4
6
5
HFD vs SD
B
HFD vs SD
HFD-R vs HFD
Actinobacteria
Bacteroidetes
Verrucomicrobiae
Firmicutes
Enterorhabdus ASV94
Bifidobacterium ASV5
Muribaculaceae* ASV3
Akkermansia ASV2
Lactobacilus ASV120
Dubosiella ASV1
log2FC
Tyrosine metabolism
Tropane, piperidine, pyridine alkaloid biosynthesis
Legionellosis
Fructose and mannose metabolism
Carbapenem biosynthesis
Arginine and proline metabolism
log2FC
C
Cecum
16 wks
68
24 wks
67
24
51
HFD-R vs HFD
16
35
32
HFD vs SD
D
Enterorhabdus ASV94
Intestinimonas ASV93
Lachnoclostridium ASV89
Lachnoclostridium ASV30
Lachnoclostridium ASV142
Lachnospiraceae* ASV67
Lachnospiraceae* ASV179
Lachnospiraceae* ASV177
Lachnospiraceae* ASV105
Lachnospiraceae_NK4A136_group ASV59
Lachnospiraceae_NK4A136_group ASV55
Lachnospiraceae_NK4A136_group ASV28
Lachnospiraceae_FCS020_group ASV178
Lachnospiraceae_FCS020_group ASV162
Lachnospiraceae_UCG-006 ASV57
Lactobacilus ASV120
Muribaculaceae* ASV153
Muribaculaceae* ASV3
Adlercreutzia ASV42
Acetatifactor ASV164
Anaeroplasma ASV72
Acetatifactor ASV145
A2 ASV108
Anaerotruncus ASV196
Bifidobaterium ASV5
Turicibacter ASV16
Family_XIII* ASV47
Family_XIII_AD3011_group ASV111
Ruminococcaceae* ASV64
Ruminiclostridium_5 ASV137
Peptococcaceae* ASV138
NA* ASV81
NA* ASV140
Erysipelatoclostridium ASV15
Dubosiella ASV1
log2FC
Xylene degradation
Tyrosine metabolism
Tropane, piperidine and pyridine alkaloid biosynthesis
Thyroid hormone synthesis
Staphylococcus aureus infection
Serotonergic synapse
Salivary secretion
Riboflavin metabolism
Protein processing in endoplasmic reticulum
Phosphatidylinositol signaling system
Phenylalanine, tyrosine and tryptophan biosynthesis
Penicillin and cephalosporin biosynthesis
Pancreatic secretion
Novobiocin biosynthesis
Nonribosomal peptide structures
Meiosis − yeast
Lipoarabinomannan (LAM) biosynthesis
Legionellosis
Isoquinoline alkaloid biosynthesis
Insulin secretion
Insulin resistance
Gastric acid secretion
Fructose and mannose metabolism
Flavone and flavonol biosynthesis
Ethylbenzene degradation
Endocrine and other factor-regulated Ca reabsorption
Dopaminergic synapse
Dioxin degradation
Cocaine addiction
Chagas disease (American trypanosomiasis)
cGMP−PKG signaling pathway
Cardiac muscle contraction
Carbon fixation pathways in prokaryotes
Carbon fixation in photosynthetic organisms
Carbapenem biosynthesis
cAMP signaling pathway
Bile secretion
Arginine and proline metabolism
Amphetamine addiction
alpha−Linolenic acid metabolism
Aldosterone−regulated sodium reabsorption
Aldosterone synthesis and secretion
Alcoholism
African trypanosomiasis
Adrenergic signaling in cardiomyocytes
Adipocytokine signaling pathway
log2FC
E
Colon
16 wks
53
24 wks
56
42
28
HFD-R vs HFD
31
25
17
HFD vs SD
F
Candidatus_Stoquefichus ASV98
Enterorhabdus ASV94
Lachnoclostridium ASV89 ASV142
Clostridium_sensu_stricto_1 ASV73
Anaeroplasma ASV72
Ruminococcaceae* ASV64
Lachnospiraceae_NK4A136_group ASV59 ASV55
Family_XIII* ASV44
Muribaculaceae* ASV28
Lachnospiraceae* ASV47
Turicibacter ASV3 ASV153
NA* ASV179
Erysipelatoclostridium ASV16
ASF356* ASV81
ASV15
Acetatifactor ASV146
Bifidobaterium ASV145
Lactobacilus ASV5
Family_XII_AD3011-group ASV120
A2 ASV111
Anaerotruncus ASV108 ASV106
log2FC
Tyrosine metabolism
Tropane, piperidine, pyridine alkaloid biosynthesis
Serotonergic synapse
Penicillin and cephalosporin biosynthesis
Lysine biosynthesis
Legionellosis
Isoquinoline alkaloid biosynthesis
Insulin resistance
Dopaminergic synapse
Cysteine and methionine metabolism
Cocaine addiction
Chagas disease (American trypanosomiasis)
Carbon metabolism
Carbohydrate digestion and absorption
Carbapenem biosynthesis
Arginine and proline metabolism
Amphetamine addiction
Alcoholism
Adipocytokine signaling pathway
log2FC

Figure S2. Dietary reversal shifts microbial taxa signature in obese PN mice. Analysis to identify differentially abundant bacteria with DESeq2 between HFD versus SD at 16 weeks of age or between HFD versus SD, HFD-R versus HFD, and HFD-R versus SD at 24 weeks of age in (**A**) ileum, (**C**) cecum, and (**E**) colon (adjusted *P*-value<0.05). Overlap in gut microbial taxa (left) and KEGG pathways (level 1, right; HFD versus SD, orange; HFD-R versus HFD, purple) driving differences between dietary fat at 24 weeks in (**B**) ileum, (**D**) cecum, and (**F**) colon presented as bar plots of $\log_2$ fold-change ($\log_2$FC). ASVs are listed with corresponding genus or family (*) level. Taxa shared among ileum, cecum, and colon, as well as pathways shared by ileum, cecum, colon, and fecal pellets (**Figure 3**), are listed in red text. Multiple t-testing was used for pathway comparisons and significant pathways were identified by FDR adjusted *P*-value <0.05.

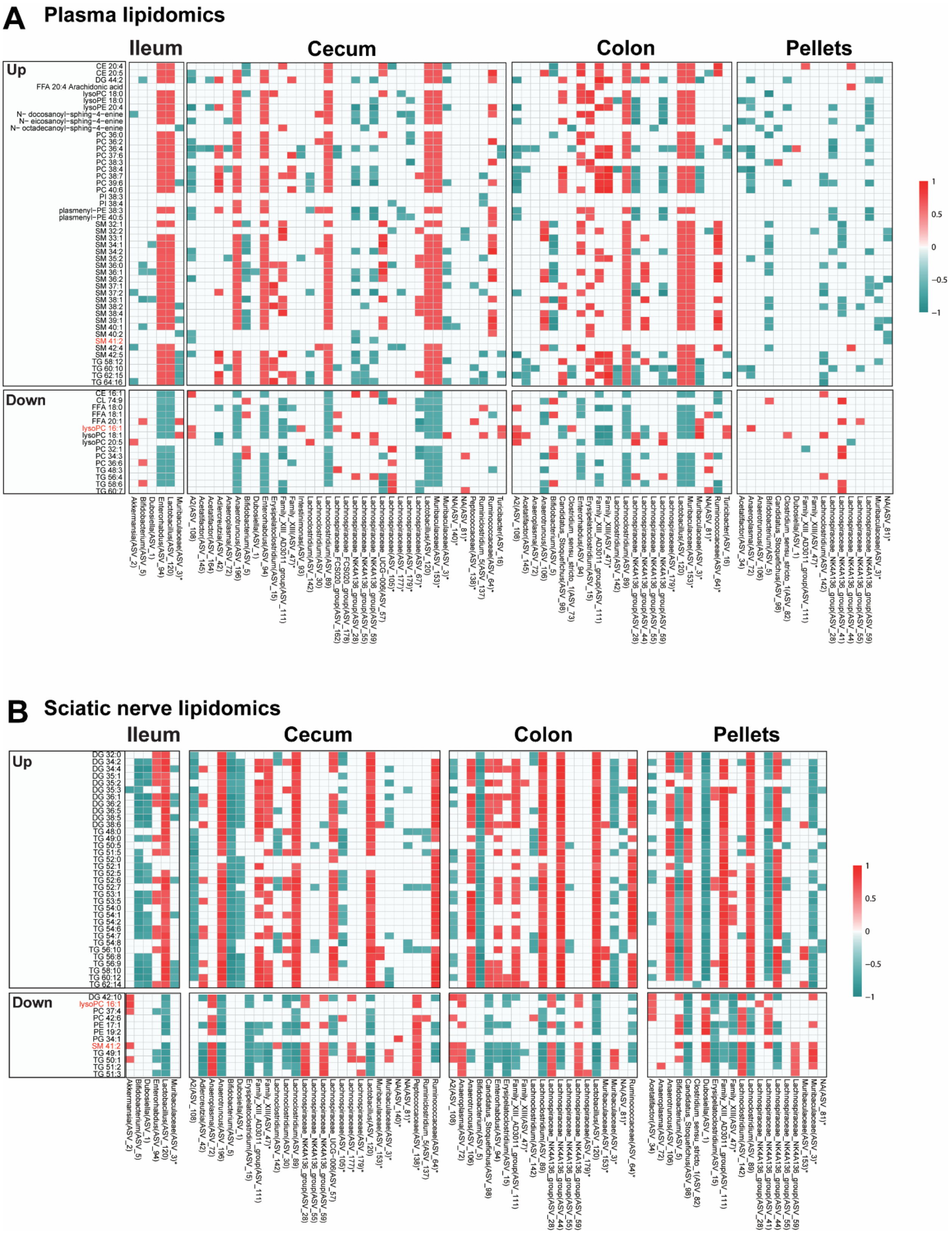
A
Plasma lipidomics
Ileum
Cecum
Colon
Pellets
Up
CE 20:4
CE 20:5
DG 44:2
FFA 20:4 Arachidonic acid
lysoPC 18:0
lysoPE 18:0
lysoPE 20:4
N- docosanoyl-sphing-4-enine
N- eicosanoyl-sphing-4-enine
N- octadecanoyl-sphing-4-enine
PC 36:0
PC 36:2
PC 36:4
PC 37:6
PC 38:3
PC 38:4
PC 38:7
PC 39:6
PC 40:6
PI 38:3
PI 38:4
plasmenyl-PE 38:3
plasmenyl-PE 40:5
SM 32:1
SM 32:2
SM 33:1
SM 34:1
SM 34:2
SM 35:2
SM 36:0
SM 36:1
SM 36:2
SM 37:1
SM 37:2
SM 38:1
SM 38:2
SM 38:4
SM 39:1
SM 40:1
SM 40:2
SM 41:2
SM 42:4
SM 42:5
TG 58:12
TG 60:10
TG 62:15
TG 64:16
Down
CE 16:1
CL 74:9
FFA 18:0
FFA 18:1
FFA 20:1
lysoPC 16:1
lysoPC 18:1
lysoPC 20:5
PC 32:1
PC 34:3
PC 36:6
TG 48:3
TG 56:4
TG 58:6
TG 60:7
Akkermansia(ASV_2)
Bifidobacterium(ASV_5)
Dubosiella(ASV_1)
Enterorhabdus(ASV_94)
Lactobacillus(ASV_120)
Muribaculaceae(ASV_3)*
1
0.5
0
−0.5
−1
B
Sciatic nerve lipidomics
Ileum
Cecum
Colon
Pellets
Up
DG 32:0
DG 34:2
DG 34:4
DG 35:1
DG 35:2
DG 35:3
DG 36:1
DG 36:2
DG 36:5
DG 38:5
DG 38:6
TG 48:0
TG 49:0
TG 50:5
TG 51:5
TG 52:0
TG 52:1
TG 52:5
TG 52:6
TG 52:7
TG 53:1
TG 53:5
TG 54:0
TG 54:1
TG 54:2
TG 54:6
TG 54:7
TG 54:8
TG 56:10
TG 56:8
TG 56:9
TG 58:10
TG 60:12
TG 62:14
Down
DG 42:10
lysoPC 16:1
PC 37:4
PC 42:6
PE 17:1
PE 19:2
PG 34:1
SM 41:2
TG 49:1
TG 50:1
TG 51:2
TG 51:3
Akkermansia(ASV_2)
Bifidobacterium(ASV_1)
Dubosiella(ASV_1)
Enterorhabdus(ASV_94)
Lactobacillus(ASV_120)
Muribaculaceae(ASV_3)*
1
0.5
0
−0.5
−1

Figure S3. Full correlation analysis for microbial communities to plasma and sciatic nerve lipidomics. Full Spearman's correlation analysis heatmaps (FDR<0.05) of relative abundance of PN-associated gut microbiota sensitive to dietary fat at 24 weeks of age with (**A**) plasma DALs and (**B**) sciatic nerve DALs [27], which are increased (Up) or decreased (Down) in in the overlapping DALs between HFD vs SD and HFDR vs HFD in ileum, cecum, colon, and fecal pellets. Correlation scale (red, positive; green, negative) is the same for (A) and (B). CE, cholesteryl esters; CL, cardiolipins; DG, diglycerides; FFA, free fatty acids; lysoPC, lysophosphatidylcholines; lysoPE, lysophosphatidylethanolamines; PC, phosphatidylcholines; PE, phosphatidylethanolamines; PI, phosphatidylinositols; pPE, plasmenyl-phosphatidylethanolamines; SM, sphingomyelins; TG, triglycerides.

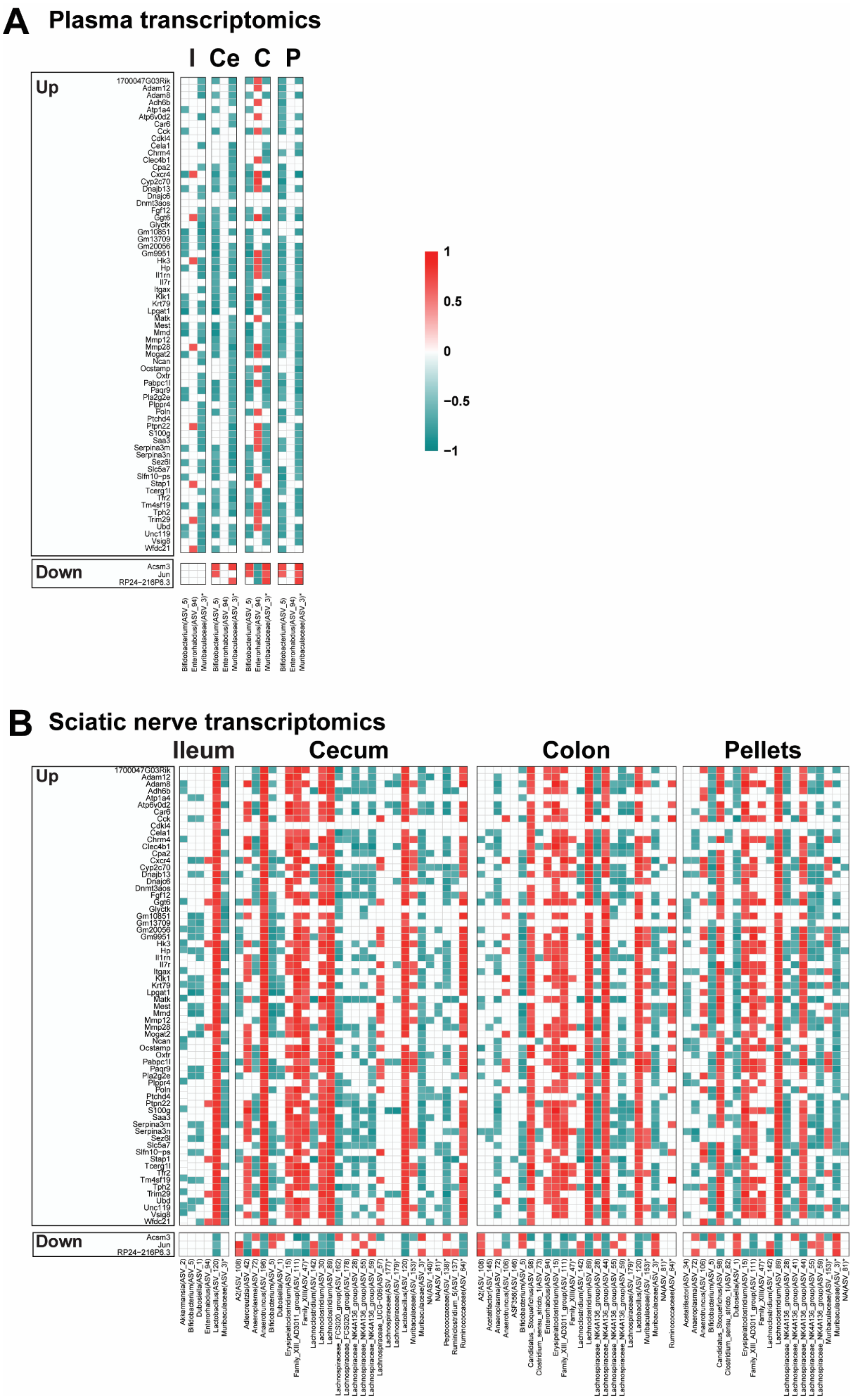
A
Plasma transcriptomics
I
Ce
C
P
Up
1700047G03Rik
Adam12
Adam8
Adh6b
Atp1a4
Atp6v0d2
Car6
Cck
Cdkl4
Cela1
Chrm4
Clec4b1
Cpa2
Cxcr4
Cyp2c70
Dnajb13
Dnajc6
Dnmt3aos
Fgf12
Ggt6
Glyctk
Gm10851
Gm13709
Gm20056
Gm9951
Hk3
Hp
Il1rn
Il7r
Itgax
Klk1
Krt79
Lpgat1
Matk
Mest
Mmd
Mmp12
Mmp28
Mogat2
Ncan
Ocstamp
Oxtr
Pabpc1l
Paqr9
Pla2g2e
Plppr4
Poln
Ptchd4
Ptpn22
S100g
Saa3
Serpina3m
Serpina3n
Sez6l
Slc5a7
Slfn10-ps
Stap1
Tcerg1l
Tlr2
Tm4sf19
Tph2
Trim29
Ubd
Unc119
Vsig8
Wfdc21
Down
Acsm3
Jun
RP24-216P6.3
Bifidobacterium(ASV_5)
Enterorhabdus(ASV_94)
Muribaculaceae(ASV_3)*
1
0.5
0
−0.5
−1
B
Sciatic nerve transcriptomics
Ileum
Cecum
Colon
Pellets
Up
Down

Figure S4. Full correlation analysis for microbial communities to plasma and sciatic nerve transcriptomics. Full Spearman's correlation analysis heatmaps (FDR<0.05) of relative abundance of PN-associated gut microbiota sensitive to dietary fat at 24 weeks of age with (**A**) plasma DEGs (n=11) or (**B**) sciatic nerve DEGs [27] (n=11), which are upregulated (Up) or downregulated (Down) in the overlapping DEGs between HFD vs SD and HFDR vs HFD in ileum, cecum, colon, and fecal pellets. Correlation scale (red, positive; green, negative) is the same for (A) and (B). C, colon; Ce, cecum; I, ileum; P, pellets.

SUPPLEMENTARY TABLES

Table S1. Study microbiome samples. Type (ileum, cecum, colon, fecal pellets), number, and time points for collected microbiome samples.

Sample	Age (weeks)	Group	n
**Ileum (38)**	16	SD	8
		HFD	6
	24	SD	8
		HFD	8
		HFD-R	8
**Cecum (38)**	16	SD	8
		HFD	6
	24	SD	8
		HFD	8
		HFD-R	8
**Colon (36)**	16	SD	8
		HFD	6
	24	SD	6
		HFD	8
		HFD-R	8
**Fecal pellets (247)**	8	SD	16
		HFD	16
		HFD-R	8
	10	SD	15
		HFD	16
		HFD-R	8
	12	SD	16
		HFD	16
		HFD-R	6
	16	SD	16
		HFD	14
		HFD-R	8
	18	SD	8
		HFD	8
		HFD-R	7
	20	SD	8
		HFD	8
		HFD-R	8
	22	SD	7
		HFD	7
		HFD-R	7
	24	SD	8
		HFD	8
		HFD-R	8

Table S2. Beta diversity in microbiome samples. Permutational analysis of variance (PERMANOVA) of beta diversity in mouse gut bacteria responding to dietary fat content.

	**Ileum**	**Cecum**	**Colon**	**Fecal pellets**
**Age (weeks)**	*P*-values			
**8**				0.001
**16**	0.002	0.001	0.003	0.001
**18**				0.001
**24**	0.001	0.001	0.001	0.001

References

27. O'Brien, P.D., et al., Integrated lipidomic and transcriptomic analyses identify altered nerve triglycerides in mouse models of prediabetes and type 2 diabetes. Dis Model Mech, 2019.
